# Bilateral versus ipsilateral neck dissection in oral and oropharyngeal cancer with contralateral cN0 neck

**DOI:** 10.1007/s00405-020-06043-2

**Published:** 2020-05-24

**Authors:** Andreas Knopf, Sven Jacob, Henning Bier, Elias Q. Scherer

**Affiliations:** 1 Department of Otorhinolaryngology-Head and Neck Surgery, University Medical Centre Freiburg, Killianstr. 5, 79106 Freiburg, Germany; 2 Hals-Nasen-Ohrenklinik, Klinikum rechts der Isar, Technische Universität München, Ismaninger Str. 22, 81675 München, Germany; 3 Hals-Nasen-Ohrenklinik, Agaplesion Diakonieklinikum Rotenburg, Elise-Averdieck Str. 17, 27356 Rotenburg, Germany

**Keywords:** SCC, Oropharynx, Oral cavity, Elective neck dissection

## Abstract

**Objective:**

Contralateral elective neck dissection (cEND) in oral and oropharyngeal squamous cell cancer (OC/OPC) is still a matter of debate. The current study analyzed the outcome in OC/OPC patients with/without cEND.

**Methods:**

OC/OPC patients (*n* = 471) were diagnosed with contralateral N0 after CT/MRI-scan combined with neck ultrasound. Clinico-pathological features were analyzed using Chi-square/Fisher exact/Student’s *t* test. Survival rates were calculated using Kaplan–Meier and log-rank test. Prognostic variables were evaluated by Cox regression. Primary/secondary endpoints were overall/recurrence-free survival (OS/RFS).

**Results:**

Pre-therapeutic imaging revealed a significantly over-staged N-status (*p* = 0.01), while occult contra-lateral N + was diagnosed in one patient only (0.4%). OC patients did not show differences in OS/RFS between the groups (ipsi- vs. bi-lateral). There was a strong tendency towards a better OS in OPC patients who underwent ipsi-lateral ND (*p* = 0.07). Cox-regression demonstrated that only tumor recurrence was associated with a fivefold increased risk of recurrence-associated death (*p* < 0.0001) that referred to a significant higher recurrence rate at primary tumor site (rT +) and increased distant metastatic outgrowth in OPC who underwent bi-lateral neck dissection (*p* = 0.03). While RFS of any cause (rT + /rN + /rM +) was significantly better in OPC with ipsi-lateral ND (*p* < 0.05), RFS of contralateral lymph node recurrence (rN2c) was comparable in both groups.

**Conclusion:**

END of the contralateral cN0 neck is not correlated by an increased RFS or OS. Standard imaging techniques including CT/MRI scan and neck ultrasound warrant watchful waiting for neck dissection of the contralateral cN0 neck.

## Introduction

Head and neck squamous cell carcinoma (HNSCC) is the sixth most common cancer worldwide accounting for approximately 500,000 newly diagnosed cases and 300,000 deaths every year [1, 2]. The majority of HNSCC originate in the oro-/hypopharynx, larynx, and oral cavity [3]. Beside HPV status in OPC, patient’s prognosis is inherently associated with the T-, N-, M-status [3, 4]. The presence of cervical lymph node metastasis is the most significant prognostic factor for oral and oropharyngeal squamous cell carcinoma (OC, OPC) [5]. The risk of cervical lymph node metastasis depends on tumor location and size [6, 7]. Selective or radical neck dissection is currently considered as the gold standard in the surgical treatment of lymph node-positive (N +) oral and oropharyngeal cancer [8–10].

Ipsilateral neck dissection in clinically node-negative (N0) individuals has been a matter of debate for the last 5 decades. Surgical options for addressing the N0 neck include elective neck dissection or watchful waiting with therapeutic neck dissection for nodal relapse. However, treatment with elective neck dissection (END) at the time of primary tumor resection has proven to be associated with an increased overall (OS) and recurrence-free survival (RFS) [7, 11–13]. Therefore, END of the ipsilateral cN0 neck can be considered a standard procedure for OC and OPC. There are still controversies whether to perform END of the contralateral cN0 neck. Only few publications address this issue in OC and OPC with no definite evidence in terms of OS and RFS [14–16].

This retrospective study analyzed if END of the contralateral N0 neck in oral and oropharyngeal cancer has an improved OS and/or RFS and whether this effect is dependent on tumor size and laterality of the primary site.

## Patients and methods

### Patient selection and statistical analysis

A total of 521 consecutively selected, surgically treated OC/OPC patients who underwent ipsilateral and bilateral neck dissection in OPC and OC during a period of 10 years (01.01.2001–31.12.2011) were screened. All patients underwent otolaryngological examination and clinical evaluation of primary tumor site that was supplemented by neck CT and/or MRI imaging. CT and/or MRI imaging as well as head and neck ultrasound were performed to exclude lymph node involvement. Patients suspicious for contralateral lymph node manifestation after primary staging were excluded. N0 neck was diagnosed when none of the imaging techniques visualized pathological findings. Irregularly shaped lymph nodes, lymph nodes with aspect ratio < 1.5:1, lymph nodes with a maximum diameter > 1 cm, and signs of intra-nodal necrosis were defined being cN + . Patients with recurrent disease, distant metastasis at the time of diagnosis, and patients with treatment referring to study protocols were excluded. Tumor samples were histologically reviewed by at least two experienced pathologists. Dysplasia, carcinoma in situ, and other histologic subtypes such as adenocarcinoma were excluded from the study.

A total of 471 patients with contralateral N0-neck were included in the current study. Clinical parameters and survival data were retrospectively collected: age, sex, TNM-status, grading, treatment modalities, recurrence, and death/loss to follow-up. 7th UICC classification system was used to avoid clinical pre-interpretation of N-status that biases further regression analysis. All patients underwent standard therapeutic protocols referring to international guidelines, including adjuvant radiotherapy in all node-positive individuals, and chemotherapeutic therapy escalation in extra-capsular extension and/or insufficient R-status. Patients who underwent therapeutic protocols referring to study cohorts were excluded. Patients with lacking data, incomplete staging, and refused/not finished surgical and/or conservative treatment (radio-/chemotherapy) were excluded from survival analysis. The mean follow-up time was 66 months. The overall cohort was divided into patients who underwent ipsi-lateral neck dissection and patients with bi-lateral neck dissection. Pre- and postoperative lymph node involvement was categorized with respect of subsequent surgical procedure into (i) none, (ii) ipsi-lateral metastasis, and (iii) bi-lateral metastasis. Midline-reaching tumors were defined by macroscopic tumor extension ≤ 10 mm to the midline and midline-crossing tumors by contralateral extension.

Differences between the groups were analyzed using the Chi-square test and Fisher exact test for categorical, and the unpaired Student’s *t* test for continuous variables. As main endpoints, the overall (OS) and recurrence-free survival (RFS) were assessed measuring the time from treatment to death of any cause and recurrence. The oncological outcome of elective contra-lateral neck dissection was exclusively analyzed in patients who were not suspicious for contra-lateral lymph node involvement. Overall survival and recurrence-free survival were comprehensively analyzed in oral and oropharyngeal carcinomas. To assess direct implication of wait and scan concept in contralateral neck-negative individuals, recurrence-free survival was exclusively analyzed for lymph node recurrence. Patients with distant metastasis or recurrence at primary tumor site were excluded from the analysis of recurrence-free survival. Survival rates and curves were calculated and illustrated by the Kaplan–Meier method and further analyzed by the log-rank test for univariate analysis. Variables that revealed prognostic or effect-modifying potential on the outcome as suggested by univariate analysis were subsequently evaluated by the proportional Cox regression for multivariate analysis. *p* values < 0.05 were considered statistically significant. Statistical analysis was done using SPSS (SPSS Inc., Chicago, IL).

The local ethical committee approved the study (191/15 s).

## Results

### Patient/tumor characteristics and survival in oral cancer (OC)

A total of 142 consecutively treated patients with OC were included in the current study. The majority of patients was treated with bi-lateral neck dissection (*n* = 83), while 59 patients underwent ipsi-lateral neck dissection. The mean age at diagnosis was 57. There was a striking male predominance, but no differences between the groups with respect to gender and age (Table 1).

**Table 1:** Clinico-pathological characteristics and therapeutic strategy in oral cancer

	Ipsi-lat. ND	Bilat. ND	*p* value
*n*	59	83	
Age (years)			0.91
Median	56.0	57.00	
Mean ± SD	57 ± 13	57 ± 11	
Sex, *n* (%)			0.64
Male	41 (70)	61 (74)	
Female	18 (30)	22 (26)	
Location, *n* (%)			
Cheek	6 (10)	3 (4)	0.34
Buccoalveolar sulcus	1 (2)	4 (5)	
Mouth floor	12 (20)	47 (57)	
Tongue	40 (68)	29 (35)	
Laterality, *n* (%)			< 0.0001
Lateral	47 (80)	40 (48)	
Mid-line reaching	11 (18)	23 (28)	
Mid-line crossing	1 (2)	20 (24)	
pT-status, *n* (%)			0.07
T1	35 (59)	38 (46)	
T2	18 (31)	32 (39)	
T3	3 (5)	7 (8)	
T4	3 (5)	6 (7)	
pN-status, *n* (%)			0.38
None	34 (58)	51 (61)	
Ipsi-lateral	25 (42)	32 (39)	
Bi-lateral	0	0	
Grading, *n* (%)			0.18
G1	6 (10)	12 (15)	
G2	34 (58)	53 (64)	
G3	18 (31)	18 (22)	
G4	1 (2)	0	
R-status, *n* (%)			0.60
R0	56 (95)	81 (98)	
R1	3 (5)	1 (1)	
R2	0	0	
Rx	0	1 (1)	
ECE, *n* (%)			0.29
Positive	1 (2)	4 (5)	
Primary tumor resection, *n* (%)			< 0.0001
Transoral	53 (90)	49 (59)	
Transmandibular	6 (10)	34 (41)	
Adjuvant therapy, *n* (%)			0.02
None	29 (50)	26 (31)	
C/RT	30 (51)	57 (69)	

Disease related data of the analyzed study cohort. The clinical indication contralateral END was done due to laterality and T-status

*ECE* extra-capsular extension

OC who underwent bi-lateral neck dissection showed a significantly higher proportion of midline-reaching/crossing tumors (*p* < 0.0001) and a tendency towards a higher T-status (*p* = 0.07) when compared with patients who were treated with ipsi-lateral neck dissection (Table 1). In OC, there were no differences in the pN-status with respect to performed neck dissection. Eighty-five OC patients (60%) showed N0 neck, while ipsilateral lymph node involvement was diagnosed in 57 patients (40%). None of the OC patients showed occult contralateral metastasis. Tumor grading revealed G2/3-status in the vast majority of OC. One hundred thirty-seven patients (97%) were resected *in sano* without differences between the groups (Table 1). Histological examination revealed extra-capsular extension (ECE) in only five patients (4%) (Table 1).

Surgical strategies differed significantly in ipsi- and bi-lateral-treated OC patients. While ipsi-lateral neck dissection usually supplemented transoral resection, bi-lateral neck dissection showed an increase in transmandibular approaches (*p* < 0.0001; Table 1). Adjuvant radiotherapy was recommended in patients with increased T-status (≥ T3), lymph node positivity, or insufficient R-status. Adjuvant radio-chemo-therapy was applied in patients with N + neck with extracapsular extension or insufficient R-status. Adjuvant radio(chemo)therapy was performed in 30 patients (51%) after ipsi-lateral neck dissection and 57 patients (69%) after bi-lateral neck dissection (*p* = 0.02; Table 1).

**Fig. 1:**
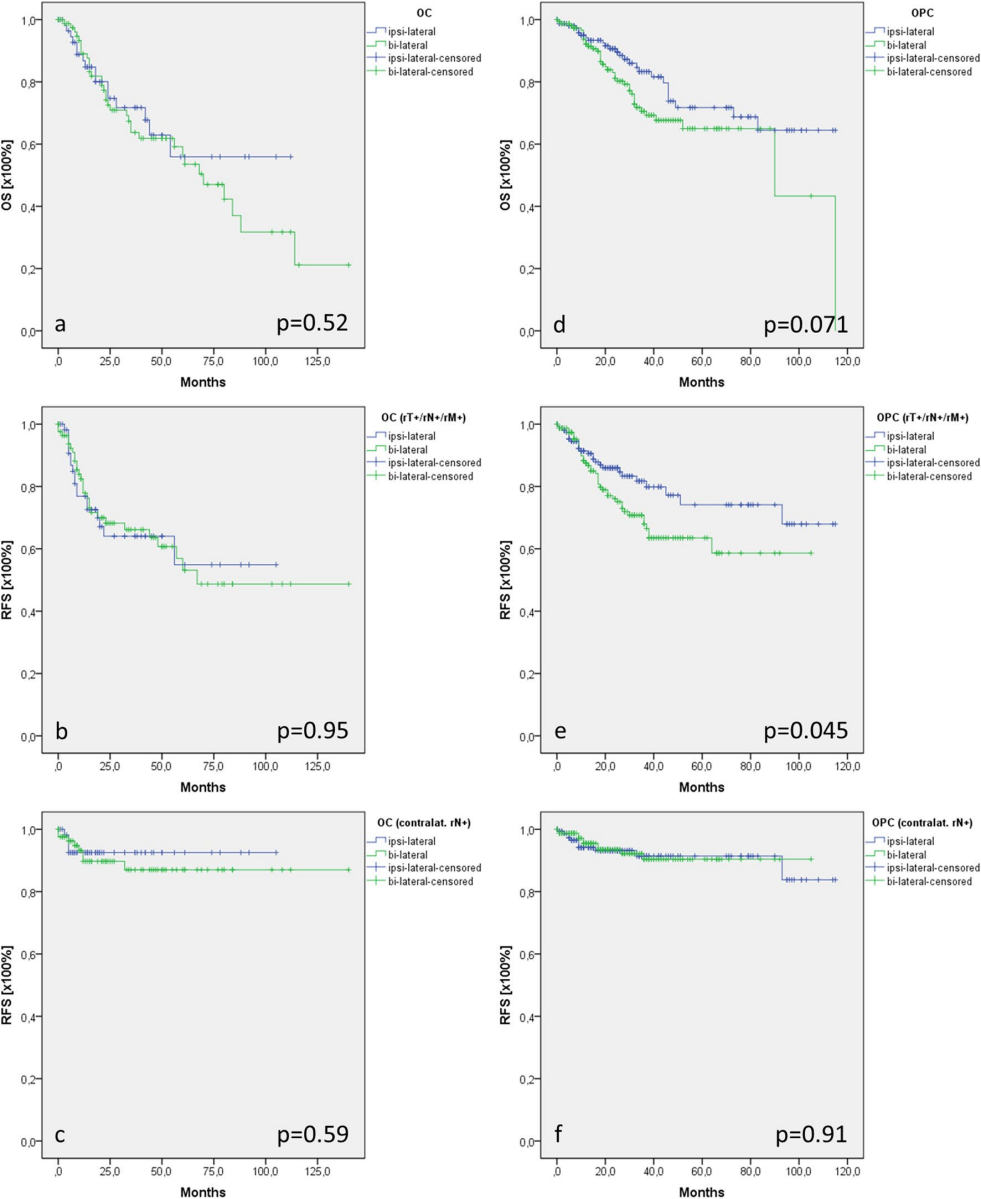
Oncological outcome of oral and oropharyngeal carcinoma. In OC there were no differences in the OS (**a**), RFS (of any case, **b**), and RFS of contralateral lymph node involvement (**c**). In contrast, in OC there was a tendency towards a better OS of patients who underwent ipsi-lateral neck dissection (**d**). Significant better RFS (of any case, **e**) was demonstrated for patients who were treated with ipsi-lateral neck dissection when compared with patients who underwent bi-lateral neck dissection, while no differences could be observed in the RFS of contra-lateral lymph node disease (**f**)

Analysis of OS and RFS in OC patients showed a mean OS of 74 months in patients who underwent ipsi-lateral neck dissection and 72 months in patients with bi-lateral neck dissection (*p* = 0.52; Fig. 1a). Additionally, RFS in tumor recurrence of any cause (rT + /rN + /rM +) as well contralateral lymph node recurrence (rN2c) did not show differences between the groups (*p* = 0.95; *p* = 0.59; Fig. 1b, c).

### Patient/tumor characteristics and survival in oropharyngeal cancer (OPC)

Three hundred twenty-nine patients with consecutively treated OPC were included. A total of 165 OPC patients underwent ipsi-lateral neck dissection, while 164 patients were treated with bi-lateral neck dissection. In comparison to OC, OPC patients were slightly older demonstrating a mean age at diagnosis of 59/60 years. In agreement with OC patients, there was a notable male predominance. No differences were identified between the groups respecting of gender and age (Table 2).

**Table 2:** Clinico-pathological characteristics and therapeutic strategy in oropharyngeal cancer

	Ipsi-lat. ND	Bilat. ND	*p* value
*n*	165	164	
Age (years)			0.33
Median	59.00	59.00	
Mean ± SD	60 ± 10	59 ± 9	
Sex, *n* (%)			0.73
Male	131 (79)	128 (78)	
Female	34 (21)	36 (22)	
Location, *n* (%)			0.03
Tonsil	109 (66)	86 (52)	
Soft palate	14 (9)	12 (7)	
Uvula	2 (1)	20 (12)	
Tongue base	24 (15)	33 (20)	
Lat. pharyngeal wall	8 (5)	2 (1)	
Dorsal pharyngeal wall	1 (1)	4 (2)	
Vallecula	7 (4)	7 (4)	
Laterality, *n* (%)			< 0.0001
Lateral	135 (82)	95 (58)	
Mid line reaching	19 (12)	31 (19)	
Mid line crossing	11 (7)	38 (23)	
pT-status, *n* (%)			0.004
T1	73 (44)	51 (31)	
T2	71 (43)	79 (48)	
T3	15 (9)	22 (13)	
T4	6 (4)	12 (7)	
pN-status, *n* (%)			0.09
None	43 (26)	44 (27)	
Ipsi-lateral	122 (74)	119 (73)	
Bi-lateral	0	1	
Grading, *n* (%)			0.77
G1	4 (2)	4 (2)	
G2	68 (41)	64 (39)	
G3	92 (56)	96 (59)	
G4	1 (1)	0	
R-status, *n* (%)			0.54
R0	143 (87)	146 (89)	
R1	19 (12)	15 (9)	
R2	0	0	
Rx	3 (2)	3 (2)	
ECE, *n* (%)			0.56
Positive	23 (14)	26 (16)	
165	164		
Primary tumor resection, *n* (%)			0.001
Transoral	110 (67)	77 (47)	
Transmandibular	33 (20)	55 (34)	
Lat. pharyngotomy	22 (13)	28 (17)	
Med. pharyngotomy	0	4 (2)	
Adjuvant therapy, *n* (%)			0.56
None	30 (18)	29 (18)	
C/RT	135 (82)	135 (82)	

Disease related data of the analyzed study cohort. The clinical indication contralateral END was done due to laterality and T-status

*ECE* extra-capsular extension

In OPC, there were significant differences in the tumor location, laterality at primary tumor site, and corresponding T status. Patients who underwent bi-lateral neck dissection showed a higher percentage of midline-reaching/crossing tumors when compared with patients who were treated with ipsi-lateral neck dissection (*p* < 0.0001) that referred to a significant higher proportion of advanced tumor stages (T3/4; *p* = 0.004) and tumors originating in the uvula and base of the tongue (*p* = 0.027) (Table 2). According to OC, OPC patients did not show any differences in the pN-status with respect to performed neck dissection. Eighty-seven patients (26%) were diagnosed with N0-neck, while 241 patients (73%) showed ipsi-lateral lymph node metastases (Table 2). Occult contralateral metastasis was diagnosed in one patient (Table 2). Comparable with OC, tumor grading could be attributed to G2/3 in the majority of tumor specimen. R0-resection was achieved in 289 OPC patients (88%). In contrast to OC, a substantial proportion of OPC patients (*n* = 49) showed ECE.

In accordance with therapeutic strategies in OC, OPC patients with transmandibular approaches underwent significantly more often bi-lateral neck dissection as patients after transoral surgery (*p* = 0.001; Table 2). In both, patients with ipsi-latertal and bi-lateral neck dissection, 82% of patients underwent adjuvant treatment without differences between the groups (*p* = 0.56; Table 2).

OPC patients who underwent ipsi-lateral neck dissection (88 months) showed a strong tendency towards a better OS when compared with patients after bi-lateral neck dissection (80 months), but failed to achieve statistical significance (*p* = 0.07; Fig. 1d). Cox regression for forward selection analyzing localization at primary tumor site, laterality of the primary tumor, cT, cN, pT, pN, and tumor recurrence as disease-modifying parameters demonstrated that only tumor recurrence was associated with a fivefold increased risk of recurrence associated death (*p* < 0.0001; HR: 5.1 (95%-CI: 3.1; 8.1). Tumor recurrence occurred in 43 OPC patients (26%) with bi-lateral neck dissection and 27 OPC patients (165) who were treated with ipsi-lateral neck dissection that referred to a significant higher recurrence rate at primary tumor site (rT +) and increased distant metastatic outgrowth (rM +) (*p* = 0.033; Table 3). Subsequently, patients with ipsi-lateral neck dissection showed with a mean of 90 months a significant better RFS (of any cause) than patients with bi-lateral neck dissection (72 months; *p* = 0.045; Fig. 1e), while no differences between the groups were identified in contralateral lymph node recurrence-free survival disease (*p* = 0.91; Fig. 1f).

**Table 3:** Differential localization of tumor recurrence in oropharyngeal carcinoma

	Ipislat. ND	Bilat. ND	*p* value
*n*	165	164	
rT + /rN + /rM +	27 (16)	43 (26)	0.033
rT +	15 (9)	19 (12)	
rN +	11 (7)	10 (6)	
rM +	11 (7)	19 (12)	

Tumor recurrence in OPC who underwent bi-lateral neck dissection referred to a significant higher recurrence rate at primary tumor site (rT +) and increased distant metastatic outgrowth

### Preoperative and postoperative assessment of the N-status

Primary tumor site was assessed by otolaryngological examination and neck CT or MRI. Lymph node involvement was additionally analyzed via head and neck ultrasound. Neck imaging revealed N0-status in a third of our patients; ipsi-lateral lymph node involvement was postulated in two-third, respectively. With respect to histopathological examination, there was a slight, but significant pre-operative over-staging (*p* = 0.01, Table 2). Occult contra-lateral lymph node metastasis was diagnosed in one patient representing 0.3% of the overall cohort and 0.4% of patients who underwent contralateral END (Table 4).

**Table 4:** Preoperative and postoperative assessment of the N-status

	Pre-operative	Post-operative	*p* value
N-status			
None	157 (33)	172 (37)	0.01
Ipsi-lateral	314 (67)	298 (63)	
Bi-lateral	0	1	

All patients underwent CT/MRI scan and neck ultrasound of neck lymph nodes. Lymph node positivity was diagnosed due to pathological finding in any technique. There was a slight but significant over-staging of pre-operative imaging techniques. Occult contra-lateral lymph node metastasis was diagnosed in one patient representing 0.3% of the overall cohort and 0.4% of patients who underwent contra-lateral END

## Discussion

Despite significant advances in HNSCC cancer treatment, 5-year OS is less than 50% due to both local relapse and development of distant metastases [17]. Particularly, recurrence at primary tumor site and/or the lymphatic basin represents the most important therapeutic failures. Presence of cervical lymph node metastasis at initial diagnosis is the most significant prognostic factor for oral and oropharyngeal squamous cell carcinoma (SCC) [5]. (Table 4) However, there is no world-wide gold standard in the pre-therapeutic estimation of lymph node involvement in OC and OPC. High-resolution B-mode ultrasound, (positron-emission-) computed tomography, and magnetic resonance imaging demonstrate different diagnostic sensitivities and specificities, in particular with respect to different analyzed study cohorts, that makes techniques difficult to compare. In OC and OPC with clinically negative neck occult metastasis have been demonstrated in 20–44% of patients [6]. Because there is general agreement that END is indicated when the risk of occult metastases exceeds 15–20%, most patients with clinically N0 undergo END. This approach was thought to result in overtreatment of many patients. On the other hand, salvage rates for patients with regional recurrent disease of even early-stage OC and OPC SCC are extremely low, regardless of the initial size of the lesion, the status of the cervical nodes, and the treatment used for the recurrence [18]. Once regional recurrence appears, prognosis is poor and there are few long-term survivors. Obviously, it is the aim of head and neck surgeons to prevent regional recurrences using the best possible resection and necessary adjuvant therapy even in early-stage OC and OPC. D’Cruz et al. demonstrated significantly better OS and RFS after ipsilateral END when compared with their untreated counterparts [13]. However, there is still controversy whether to perform END in the contralateral node-negative neck. The current study analyzed the oncological outcome of OC/OPC patients who underwent ipsi- and bi-lateral neck dissection and who were diagnosed with contra-lateral N0 neck. Detailed knowledge about the contralateral metastatic risk of OC and OPC is of eminent relevance whether to treat or not the contralateral neck. Treatment options for the clinically node-negative neck are END, postoperative radiotherapy and wait and scan. However, prospective trials that differentially analyze the outcome of wait and scan, contralateral radiotherapy, and elective neck dissection in contralateral node-negative individuals are still missing. END represents both, therapeutic and diagnostic procedure, while postoperative radiotherapy is only a therapeutic procedure and ‘wait and scan’ is none of both. Prognosis of contralateral neck metastasis (N2c) is poor but similar to ipsilateral metastasis (N2b) in OC and OPC. It has been shown that negative prognosis correlates stronger with the number of metastatic lymph nodes involved rather than laterality of the involved nodes [19]. In the literature, the incidence of occult contralateral metastasis in OC and OPC is estimated to be 4 and 16% [15, 16]. The risk of occult contralateral metastasis rises with tumor size and location (midline/reaching) [14, 20]. Olzowy et al. investigated in their retrospective study 356 bilaterally necked OPC patients. They state that the incidence of contralateral neck metastasis is depending on tumor size and location. Authors recommend END of the contralateral cN0 neck for midline reaching/crossing tumors and for tumors staged T2 and above. Unfortunately, this study lacks to answer the question whether END of the contralateral neck has a benefit over radiotherapy in terms of OS or RFS. Furthermore, the pre-operative nodal staging (cN) is not compared to the postoperative result (pN) so there is no information about the quality of staging standards. Today, the use of diffusion-weighted imaging (DWI) in MRI results in a significant increase in the accuracy of lymph node involvement [21]. More recently, analysis of the diagnostic accuracy of ultrasound in comparison with ^(1)(8)^F-FDG-PET/CT, and fused ^(1)(8)^F-FDG-PET-MR images with DWI for the detection of cervical lymph node metastases revealed the highest diagnostic sensitivity for ultrasound [22]. The impact of head and neck CT scans to identify occult lymph node metastasis is discussed controversially with respective sensitivities ranging from 48–100% [23, 24]. Subsequently, our series demonstrated slight, but significant over-staging of N-status that refers most likely to pre-therapeutic combination of CT/MRI-scan and ultrasonographic examination of lymph node basin. Occult contra-lateral lymph node metastasis was diagnosed in one patient representing 0.3% of the overall cohort and 0.4% of patients who underwent contra-lateral END. OC patients did not show differences in the OS and RFS when comparing patients who underwent bi- or ipsi-lateral neck dissection. In contrast, there was a strong tendency towards a better OS in OPC patients who underwent ipsi-lateral neck dissection when compared with patients after bi-lateral neck dissection. Interestingly, Cox-regression for forward selection analyzing localization at primary tumor site, laterality of the primary tumor, cT, cN, pT, pN, and tumor recurrence as disease modifying parameters demonstrated that only tumor recurrence was associated with a fivefold increased risk of recurrence-associated death. Tumor recurrence in OPC who underwent bi-lateral neck dissection referred to a significant higher recurrence rate at primary tumor site (rT +) and increased distant metastatic outgrowth (rM +). Post hoc analysis demonstrated no differences in the rN + status. Subsequently, RFS of contralateral lymph node recurrence was comparable in both groups indicting no benefit of contra-lateral END in OPC patients.

We have to assume that the release of contralateral END in OC/OPC might be appropriate in patients who were diagnosed with contralateral N0 neck after combination of CT/MRI-scan and neck sonography. Prospective randomized trials have to assess potential surgical therapy de-escalation in detail.

## Conclusion

The current study of 471 oral and oropharyngeal cancer patients, who were staged with contralateral N0 neck in CT/MRI scan and neck ultrasound, investigates the impact of bilateral neck dissection on overall survival and recurrence-free survival. The major finding of the current study is that bilateral neck dissection of the node-negative contralateral neck did not improve overall survival or the recurrence free survival in OC and OPC patients. The tendency towards a better OS in OPC patients with bi-lateral neck dissection rather refers to a lower recurrence rate at primary and distant metastatic site than to beneficial application of contralateral END. Wait and scan as a reasonable attitude in contralateral node-negative neck has to be analyzed in prospective clinical trials.
